# Birth Weights in Sickle Cell Disease Pregnancies: A Cohort Study

**DOI:** 10.1371/journal.pone.0165238

**Published:** 2016-10-24

**Authors:** Daveena Meeks, Susan E. Robinson, David Macleod, Eugene Oteng-Ntim

**Affiliations:** 1 King’s College London School of Medicine, London, United Kingdom; 2 Department of Intensive Care and Anaesthetics, Royal Sussex County Hospital, Brighton, United Kingdom; 3 Department of Haematology, Guy’s and St. Thomas’ NHS Foundation Trust, London, United Kingdom; 4 London School of Hygiene and Tropical Medicine, London, United Kingdom; 5 Women’s Health Academic Centre, King's College London and King's Health Partners, St. Thomas’ Hospital, London, United Kingdom; Centre Hospitalier Universitaire Vaudois, FRANCE

## Abstract

Pregnancy in women with Sickle Cell Disease (SCD) has been linked with an increased incidence of adverse foetal outcomes when compared to women without haemoglobinopathies (HbAA). There’s a paucity of data into foetal outcomes for infants born to women with SCD. Customised growth charts have been demonstrated to be better than population-based growth charts at identifying unhealthy small babies. We analysed the mean birth weight and customised birth weight centiles of infants born to mothers with SCD versus mothers with HbAA genotype, to quantify the risk of having a smaller baby. Birth weight and birth weight centiles were analysed for 88 women with SCD (50 HbSS; 38 HbSC) and 176 controls (HbAA). Statistically significant differences were seen in the mean birth weight (P value = 0.004) and the mean birth weight centiles (P value = 0.016). We conclude that SCD is a risk factor for having a smaller baby.

## Introduction

The clinical care for Sickle Cell Disease (SCD) has vastly improved the quality and longevity of those affected. Pregnancies in this cohort, however, still carry a greater risk of unfavourable pregnancy outcomes [1]. The major types of SCD are: Sickle Cell Anaemia (HbSS), Sickle cell-Haemoglobin C disease (HbSC) and Sickle Cell β-Thalassaemia (HbSβthal) [2]. There is a paucity of data into foetal outcomes for infants born to women with SCD [3].

A meta-analysis of 26,823 SCD pregnancies (HbSS *n* = 1,523, HbSC *n* = 331, unspecified SCD genotype *n* = 24,969) showed a greater relative risk (RR) of small for gestational age (SGA) when analysed against women with normal haemoglobin (HbAA) (HbSS RR = 3.72, HbSC RR = 1.98) [1]. A Jamaican retrospective cohort of study of *n* = 118 HbSC and *n* = 110 HbAA pregnancies showed an increased rate of low birth weight (LBW) (HbSC 22.9%, HbAA 5.5%, p<0.0001) [4]. A UK nationwide cohort study of 109 SCD pregnancies (HbSS *n* = 55, HbSC *n* = 44, other SCD *n* = 11, unknown SCD genotype *n* = 3) based on data extracted from the United Kingdom Obstretric Surveillance Study (UKOSS) showed an overall higher prevalence of LBW in babies born to mothers with SCD (23.3%, p <0.001) compared to HbAA mothers, as well as an increased rate of LBW in babies born to HbSS versus HbSC mothers (HbSS 35.4%, HbSC 14%, p = 0.025) [5].

The existing data are based on population-based estimates, which do not adjust for the effect of maternal ethnicity and parity on birth weight [6,7]. The purpose of this study is to determine whether or not infants born to women with SCD are more prone to LWB, SGA and intrauterine growth restriction (IUGR) than infants born to women without SCD [2]. As far as we are aware, this study is novel in its use of customised birth weight growth charts to accurately investigate the relationship between maternal SCD and birth weight [8].

## Materials and Methods

A retrospective cohort study was carried out on the birth weights of the newborns of mothers with SCD delivering at St Thomas’ Hospital between 2008–2013. St Thomas’ Hospital is a teaching hospital in London, UK which provides specialist tertiary care to a metropolitan patient population. Every pregnant woman receiving treatment at our department is offered and can opt to undergo haemoglobinopathy screening [8,9].

We looked at the influence of maternal SCD on birth weight, with reference to the following maternal physiological factors: body mass index (BMI), ethnicity and the number of previous births; and infant outcomes: sex and gestational age at birth. Data were extracted from an electronic clinical database (Terranova Healthware). The midwife attending the delivery had recorded data on the database. These data were used to generate a customised birth weight prediction, which was compared to the infant’s actual weight at birth using the UK Gardosi Bulk Centile Calculator Version 6.6 [8]. This version of the software uses the gestation related optimal weight standard (GROW) based specifically on the UK population [10].

Data were only included from singleton pregnancies, because pregnancies involving more than one foetus are susceptible to considerable foetal growth and weight differences [8,11]. In addition, only women with diagnosed SCD were included in the study population—those with sickle cell trait (SCT) were excluded. Pregnancies in women with SCT are not predisposed to impaired birth weight, as evidenced by a recent UK cohort study [8].

Customised growth chart calculators control for healthy growth variations due to: ethnicity, parity, maternal habitus, gender and gestational age at delivery. Population-based growth charts thus do not accurately distinguish between permissible (non-pathologically) and pathologically small neonates, whereas customised growth charts do [8]. Customised growth charts have been shown to be better at identifying small babies than population-based growth charts [6]. Data were analysed using the UK Gardosi customised growth chart calculator [8]. The UK Gardosi Bulk Centile Calculator predicts the Terminal Optimal Weight (the foetus’ growth potential) by incorporating the following physiological factors: maternal ethnicity, parity, height and weight at the time of booking, gestation at delivery (determined by ultrasound analysis) and the foetus’ gender and birth weight. These variables were extracted from Healthware and entered into the UK Gardosi Bulk Centile Calculator Version 6.6 to generate birth weight centiles [8,12].

Women were matched by age at delivery, parity and ethnicity to control for birth weight variations pertaining to ethnicity and parity. Although this is a cohort study, the matching allowed the control population to be as representative of the study population in as many aspects as could be controlled for, so as to enable any differences in the results to be representative of the difference in haemoglobin status rather than normal physiology [13]. The upper and lower limits of the birth weight centiles on the customised growth chart were used to more accurately identify atypical growth. LBW is defined as weighing 2.5kg or less [8]. SGA describes neonates with a birth weight ranked at or below the 10^th^ centile for gestation [7].

The study population incorporated all eligible women with SCD and the control population included all eligible women without SCD. The SCD group was stratified according to SCD type into HbSS and HbSC. Statistical analyses were made comparing SCD group against the control group, and the stratified HbSS and HbSC groups against the controls, using Stata 14. Given the heterogeneity of SCD, the purpose of the latter was to enable specific analysis of the impact on haemoglobin status on foetal growth [14].

Statistical differences and 95% confidence intervals were generated using logistic regression to analyse the birth weight and birth weight centiles in the study and control groups. Differences in the mean maternal age, BMI, gestational age and birth weight were estimated along with their 95% confidence intervals. T-value was performed to obtain the p-value for the difference, and the null hypothesis defined as a difference being equal to 0. For variables expressed as proportions (parity, sex, birth weight centile, LBW and SGA) we performed logistic regression to identify any differences in the proportions for the SCD and HbAA group [6,7,14,15].

## Results

Birth weight and birth weight centiles were analysed for infants born to 88 women with SCD (50 with HbSS and 38 with HbSC genotype, respectively) and 176 controls (HbAA) (same ethnicity, age, parity and gestation) Table 1. Statistically significant differences were seen in mean birth weight (P value = 0.004) and mean birth weight centiles (P value = 0.016) Table 1. The mean age was 29.6 years in both the SCD group (28.5 years in HbSS; 31.0 years in HbSC groups) and control group (P value = 0.98). The mean BMI in the SCD group was 25.0 kg/m^2^ (22.5 kg/m^2^ in HbSS and 28.3 kg/m^2^ in HbSC groups) and 27.3 kg/m^2^ in the control group (P value = 0.003). There were no statistically significant differences in the rates of SGA or LBW amid the SCD and HbAA groups Table 2.

**Table 1:** Characteristics of study groups.

	SCD (n = 88)	HbSS (n = 50)	HbSC (n = 38)	HbAA (n = 176)	Difference (SCD vs. HbAA)	P value^¶^
Maternal Age (years)*	29.6 (5.6)	28.5 (5.7)	31.0 (5.1)	29.6 (5.4)	0.02 (95% CI: -1.4,1.4)	0.98
**BMI (kg/m**^**2**^)*	25.0 (6.2)	22.5 (3.2)	28.3 (7.6)	27.3 (5.5)	2.27 (95% CI: 0.79,3.75)	0.003
**Nulliparous (%)**^	53.4%	60.0%	44.7%	52.8%	0.6%	0.931
**Ethnicity (%)**^						
Black British	28.4%	30.0%	26.3%	28.4%	0	1
Black African	63.6%	66.0%	60.5%	63.6%	0	1
Black Other	8.0%	4.0%	13.2%	8.0%	0	1
Gestational age (weeks)*	38.3 (2.5)	38.1 (2.1)	38.5 (2.9)	38.7 (2.7)	0.44 (95% CI: -0.23,1.12)	0.20
**Female infant (%)**^	52.3%	62.0%	60.5%	50.6%	1.7%	0.79
Birth weight (g)*	2918 (587)	2858 (505)	2998 (678)	3176 (720)	258.4 (95% CI: 83.9,432.8)	0.004
Birth weight centile (%)*	32.8 (24.3)	33.0 (23.7)	42.2 (32.1)	42.1 (32.1)	9.4 (95% CI: 1.8,17.1)	0.016
**LBW (%)**	15.9% (14)	18.4% (9)	14.0% (5)	10.8% (19)	5.1% (95% CI: -3.8%, 14.0%)	0.239
**SGA (%)**	23.9% (21)	18.0% (9)	31.6% (12)	20.5% (36)	3.4% (95% CI: -7.3%, 14.1%)	0.526

*Values are mean (SD)

^¶^Statistically significant (P value <0.05)

^Logistic regression

SD = standard deviation *Matched variables—hence no statistically significant difference.

**Table 2:** Adjusted Odds ratios for SCD versus HbAA.

	SCD (n = 88)	HbAA (n = 176)	Adjusted Odds ratio (SCD vs. HbAA)	P value^¶^
**LBW (%)**^	15.9% (14)	10.8% (19)	1.56 (95% CI: 0.74, 3.29)	0.239
**SGA (%)**^	23.9% (21)	20.5% (36)	1.22 (95% CI: 0.66, 2.25)	0.526

^¶^Statistically significant (P value <0.05)

^Logistic regression.

The main differences between the SCD and control group were the mean birth weight and mean birth weight centile. The overall mean birth weight for the SCD pregnancies was 2918g compared to a mean birth weight of 3176g for the controls. The mean birth weight of the neonates born to SCD mothers was significantly lower at 258g below that of the HbAA counterparts (P value = 0.004). In keeping with this, the mean birth weight centile for the SCD deliveries (32.8^th^ centile) was significantly lower than the controls (42.2^nd^ centile) with a difference of 9.4 centiles (P value = 0.016) Table 1. The median birth weight was 3025g (interquartile range = 2740-3270g) for the SCD group and 3200g (interquartile range = 2890-3545g) for the controls.

The preterm birth rate (defined as birth prior to the 37^th^ week of gestation) was 10.2% (*n* = 9) for the SCD group (95% CI = 5.3%-18.7%) and 9.1% (*n* = 16) for the control group (95% CI = 5.6%-14.4%) [1]. There was no significant difference between the ethnicities of our study and comparator group; however, this was to be expected given that we matched our control group according to ethnicity Table 1.

## Discussion

### Key findings

Statistically significant differences were seen in mean birth weight (P value = 0.004) and mean birth weight centiles (P value = 0.016). Inadequate foetal development and/or premature labour can cause LBW [16]. Babies born SGA are more susceptible to disease and death *in utero* or after birth. Affected infants may have impaired growth and/or cognitive development, and in adult life may be susceptible to heart and vascular problems [17]. Pregnancies in women with SCD are at increased risk of maternal mortality, pre-eclampsia, preterm delivery and stillbirth; such complications may prevent the developing foetus achieving its growth potential *in utero* [1,2].

### Interpretation of findings

A neonate may exceed the threshold for SGA and still be growth restricted. IUGR reflects an infant who has been unsuccessful in attaining his or her personal optimal growth [13]. Our findings demonstrate that SCD deliveries are more at risk of smaller babies than non-SCD deliveries. There was no significant difference between the ethnicity, parity or age of the women in the SCD and control group, which further implicates SCD as the cause of the lower mean birth weight and mean birth weight centiles in the SCD group [7,18].

Gestational age at delivery is influenced by: maternal age at delivery, socioeconomic status, pathology, number of previous births, demographics, a history of preterm birth and smoking. Mothers of Black and Asian ethnicity have been shown to deliver earlier compared to white counterparts [19]. LBW has been linked with first-time mothers [20]. By matching our SCD population and controls, we ensured that there were no significant differences in the parity, gestational age at delivery, age or ethnicity of the women in our study population and matched controls Table 1. Variations in these physiological factors are therefore unlikely to account for the statistically lower birth weight and birth weight centiles seen in our SCD group [19].

Our SCD group had a significantly lower mean BMI than our controls Table 1. Maternal BMI is an important consideration when assessing foetal growth, in particular birth weight. Women with a diagnosis of HbSS tend to have a lower body habitus than their HbAA counterparts. Our findings are consistent with this observation [15].

Alarmingly, a number of the controls were overweight (BMI between 25 and 29.9) or obese (BMI ≥30) [21]. The BMI difference has little statistical significance to the results, as the customised growth calculator takes maternal body habitus into consideration [13]. The findings do, however, highlight a separate issue of maternal obesity in this cohort and its implications on foetal outcomes [21].

Maternal obesity during pregnancy exposes the foetus to increased morbidity and mortality in the short- and long-term, including perinatal death and being large for gestational age (LGA) (at or above the 90^th^ centile for birth weight for gestation at delivery and sex), and heart disease in later life, respectively [21,22]. Putting on weight is important during pregnancy for optimal foetal development. An Australian cohort study also using customised growth charts to quantify the relationship between increases in weight during pregnancy and birth weight demonstrated a significant relationship between extreme and insufficient increases in weight during gestation and higher rates of LGA and SGA, respectively [21]. Lower socioeconomic status has been correlated with poorer foetal outcomes. It is plausible, but unlikely, that this explains the statistical differences between the mean birth weights and mean birth weight centiles [11].

Babies born to black mothers have been shown to be more likely to be of LBW when compared to babies born to white mothers. A UK study quantified a 60% increased risk of LBW amongst black babies compared to white babies [22]. Customised growth charts adjust for ethnicity, which offers the aforementioned benefit of discerning pathologically from non-pathologically small babies [22,23].

### Strengths and limitations of this study

The uniqueness of this study is that through the use of the customised growth chart we have more accurately ascertained the relationship between SCD and birth weight. As far as we are aware, this study is novel in its use of customised birth weight growth charts to accurately investigate the relationship between maternal SCD and birth weight [8].

Both active and passive smoking during pregnancy have been linked with IUGR. There was only one smoker in our SCD population, thus it is highly improbable that the significant growth restriction seen in our results relates to smoking as opposed to SCD [10].

The customised growth chart does not adjust for maternal or gestational pathologies, for example gestational hypertension which is known to affect birth weight [8,21]. Problems with the placenta, mother and/or foetus may underpin the reason for higher rates of IUGR in patients with SCD [7,18].

### Clinical relevance and future areas of research

This study is the first to quantify the impact of SCD on birth weight and birth weight centiles using customised growth charts. The findings presented are therefore novel in that the analyses take into consideration the healthy differences, including maternal ethnic and anthropometric diversity, parity, gender and the stage of gestation at birth. Our study demonstrates that although SCD is a risk factor for smaller babies, adjustment for confounders and using the Gardosi customised growth chart still demonstrates a strong evidence of IUGR in women with SCD in pregnancy. This has important clinical implications given the association between lower birth weight and unfavourable outcomes [6,7,15].

At present, we lack intrauterine interventions to cure IUGR. The identification of IUGR facilitates strategic timing of birth (e.g. induction) to optimise the baby’s prospects. Our findings emphasise the importance of regular detailed routine non-invasive monitoring for indicators of IUGR in the management of all SCD pregnancies [10].

Our study identifies several areas for future research. Firstly, problems with the placenta may underpin the IUGR [7,18]. The precise pathophysiology underlying the propensity for smaller babies in women with SCD remains elusive. The placenta plays a pivotal role in foetal development, providing a critical gateway between mother and child to supply nutrients, oxygen and other such growth enhancing components. Impairments in the placenta may have devastating effects on the health of the unborn child. Placental abnormalities have been proposed to be the basis for many foetuses failing to achieve their optimal growth. Vaso-occlusive crisis occurring in the placenta may bring about tissue ischemia and death, thus threatening the viability of the placenta [24]. Prolonged hypoperfusion and poor oxygenation are likely to impair the growth of the developing foetus [25]. Aberrant vascular remodelling has been shown to be central to the pathophysiology of preeclampsia [26]. Preeclampsia is fourfold more likely to occur in HbSS pregnancies [1].

Asymmetrical IUGR affecting the third trimester has been seen in SCD pregnancies [27]. A study using umbilical arterial doppler assessment to assess intrauterine growth in HbSS pregnancies demonstrated a relationship between increased arterial impedance and lower birth weight. These findings highlight a relationship between uteroplacental insufficiency and maternal SCD [28]. Uncovering the mechanisms behind the IUGR would be the cornerstone to developing preventative measures and interventions to improve placental function [29].

Secondly, the impact of SCD, notably HbSS on preconception BMI and pregnancy weight gain [21]. Thirdly, the significant risk of small babies in this patient cohort may have significant lifelong developmental implications on the offspring of women with SCD. Our study therefore flags up the importance of paediatric childhood developmental follow up of the offspring of this cohort of patients, especially given the hypothesis that future adult disease may be related to early life development–the Barker and the Developmental Origins of Adult Health and Disease theories [29].

This study is the first to quantify the impact of SCD on birth weight and birth weight centiles using customised growth charts. We conclude that SCD is a risk factor for smaller babies [6].
